# Therapeutic Role of Sirtuins Targeting Unfolded Protein Response, Coagulation, and Inflammation in Hypoxia-Induced Thrombosis

**DOI:** 10.3389/fphys.2021.733453

**Published:** 2021-11-05

**Authors:** Khan Sadia, Mohammad Zahid Ashraf, Aastha Mishra

**Affiliations:** ^1^Department of Biotechnology, Jamia Millia Islamia, New Delhi, India; ^2^Council of Scientific and Industrial Research-Institute of Genomics and Integrative Biology, New Delhi, India

**Keywords:** sirtuins, thrombosis, hypoxia, inflammation, unfolded protein response

## Abstract

Thrombosis remains one of the leading causes of morbidity and mortality across the world. Many pathological milieus in the body resulting from multiple risk factors escort thrombosis. Hypoxic condition is one such risk factor that disturbs the integrity of endothelial cells to cause an imbalance between anticoagulant and procoagulant proteins. Hypoxia generates reactive oxygen species (ROS) and triggers inflammatory pathways to augment the coagulation cascade. Hypoxia in cells also activates unfolded protein response (UPR) signaling pathways in the endoplasmic reticulum (ER), which tries to restore ER homeostasis and function. But the sustained UPR linked with inflammation, generation of ROS and apoptosis stimulates the severity of thrombosis in the body. Sirtuins, a group of seven proteins, play a vast role in bringing down inflammation, oxidative and ER stress and apoptosis. As a result, sirtuins might provide a therapeutic approach towards the treatment or prevention of hypoxia-induced thrombosis. Sirtuins modulate hypoxia-inducible factors (HIFs) and counteract ER stress-induced apoptosis by attenuating protein kinase RNA-like endoplasmic reticulum kinase (PERK)/Eukaryotic translation initiation factor 2α (eIF2α) pathway activation. It prevents ER-stress mediated inflammation by targeting X-Box Binding Protein 1 (XBP1) and inhibiting nuclear factor kappa-light-chain-enhancer of activated B cells (NF-κβ) signaling through deacetylation. Sirtuins also obstruct nucleotide-binding domain, leucine-rich-containing family, pyrin domain containing 3 (NLRP3) inflammasome activation to reduce the expression of several pro-inflammatory molecules. It protects cells against oxidative stress by targeting nuclear factor erythroid 2-related factor 2 (Nrf2), glutathione (GSH), forkhead box O3 (FOXO3), superoxide dismutase (SOD), catalase (CAT), peroxisome proliferator-activated receptor gamma coactivator 1-α (PGC-1α), glucose-6-phosphate dehydrogenase (G6PD), phosphoglucomutase-2 (PGAM2), and NF-κB, to name few. This review, thus, discusses the potential role of sirtuins as a new treatment for hypoxia-induced thrombosis that involves an intersection of UPR and inflammatory pathways in its pathological manifestation.

## Introduction

Sirtuins (1–7) are a family of nicotinamide adenine dinucleotide (NAD+) dependent enzymes that catalyze post-translational modification of histone and non-histone proteins, transcription factors like p53, nuclear factor κ-light-chain-enhancer of activated B cells (NF-κB), forkhead box proteins (FOXOs), peroxisome proliferator-activated receptor gamma coactivator 1-alpha (PGC-1α), acetyl CoA, structural proteins like tubulin and DNA repair proteins such as poly ADP-ribose polymerase 1 (PARP1; Jęśko and Strosznajder, 2016). A class III deacetylases and deacylases, sirtuins have a conserved NAD+-binding and catalytic domain termed as the sirtuin core domain (Frye, 2000). Out of seven sirtuins, sirtuin-1 (SIRT1), sirtuin-6 (SIRT6), and sirtuin-7 (SIRT7) are localized mainly to the nucleus to regulate gene expression and deacetylate intracellular signaling proteins including histones (Chang and Guarente, 2014). Sirtuin-2 (SIRT2) is localized mainly to the cytoplasm but can also act as a nuclear protein and regulates the cell cycle (Chang and Guarente, 2014). Sirtuin-3 (SIRT3), sirtuin-4 (SIRT4), and sirtuin-5 (SIRT5) are exclusive to the mitochondria and regulate energy metabolism in response to the mitochondrial stress (Yang et al., 2016). Sirtuins are tissue and cell-specific regulated proteins that are activated in response to many stimuli such as nutritional and metabolic imbalance (Chang and Guarente, 2014), inflammation (Shen et al., 2009; Schug et al., 2010), hypoxia (Finley et al., 2011), aging and oxidative stress (Wang et al., 2016). In addition, sirtuins, through modulation of various pathways, contribute to the maintenance of cellular homeostasis (Ding et al., 2016; Gu et al., 2016). Researchers have demonstrated a protective role of sirtuins against inflammation (Rothgiesser et al., 2010; Kauppinen et al., 2013), oxidative stress (Wang et al., 2016), endoplasmic reticulum (ER)-stress (Shin et al., 2013), and coagulation (Breitenstein et al., 2011; Barbieri et al., 2012). Table 1 highlights sirtuins and their cellular locations along with their biological functions.

**Table 1:** Sirtuins and their biological functions in hypoxia induced oxidative stress, inflammation, thrombosis, and apoptosis.

Sirtuins (their location)	Biological function and their targets	Reference(s)
SIRT1 (Nucleus, Cytoplasm)	Anti-inflammatory response (HIF-1α, NF-κB, NLRP3, TNF-α, MCP-1, and ILs) Anti-thrombotic response (HIF-1α, PAFR, TF, and eNOS) Anti-oxidative response (Nrf2, FOXO3/ SOD2, CAT, and PGC-1α) Response against UPR mediated inflammation and apoptosis (PERK, CHOP, caspase-12, XBP1s, Smad3, and ATF4)	Lim et al., 2010; Schug et al., 2010; Kauppinen et al., 2013; Li et al., 2017 Mattagajasingh et al., 2007; Lim et al., 2010; Barbieri et al., 2012; Kim et al., 2016 Brunet et al., 2004; Nemoto et al., 2005; Wang et al., 2012; Gu et al., 2016 Aoyama et al., 2005; Wang et al., 2011; Liu et al., 2017; Prola et al., 2017; Ren et al., 2019
SIRT2 (Nucleus, Cytoplasm)	Anti-inflammatory response (NF-κB) Anti-thrombotic response (Akt) Anti-oxidative response (FOXO3a, PGC-1α, G6PD, PGAM2, and NF-κB)	Kauppinen et al., 2013 Moscardó et al., 2015 Krishnan et al., 2012; Wang et al., 2012; Kauppinen et al., 2013; Gomes et al., 2015
SIRT3 (Mitochondria)	Anti-inflammatory response (HIF-1α) Anti-thrombotic (NETs and TF) Anti-apoptotic (GRP78, PERK, IRE1, ATF6, and CHOP) Anti-oxidative response (FOXO3/SOD2, IDH, and CAT)	Bell et al., 2011; Finley et al., 2011 Gaul et al., 2018 Zhang et al., 2016 Brunet et al., 2004; Merksamer et al., 2013; Tao et al., 2014
SIRT4 (Mitochondria)	Anti-oxidative response	Singh et al., 2018
SIRT5 (Mitochondria)	Anti-oxidative response (SOD1) Anti-apoptotic response	Lin et al., 2013 Liu et al., 2013
SIRT6 (Nucleus)	Anti-oxidative response (AMPK-FOXO3α, and Nrf2) Anti-thrombotic response (HIF-α) Anti-inflammatory response against UPR (XBP1s)	Pan et al., 2016; Wang et al., 2016 Zhong et al., 2010 Bang et al., 2019
SIRT7 (Nucleus)	Anti-thrombotic response (HIF-1α and HIF-2α) Anti-inflammatory response against UPR (CHOP, XBP1s, and GRP78)	Hubbi et al., 2013 Shin et al., 2013

Hypoxia, a condition characterized by a low oxygen environment, transcriptionally induces a robust set of genes controlled by a master transcription factor named as hypoxia inducible factor (HIF) that helps the human body in adaptation to diminishing oxygen levels. However, persistent activation of hypoxia-induced genes can result in numerous pathologies. Vascular response to hypoxia contributes to the pathogenesis of several vascular wall diseases, respiratory and lifestyle disorders. A continual hypoxia stimulus in the cells generates reactive oxygen species (ROS), causing oxidative stress, which escalates inflammation and endothelial dysfunction. Both of these pathological pathways are the crucial features in life-threatening vascular disease conditions such as venous thrombosis. Additionally, hypoxia also contributes to the accumulation of misfolded proteins in ER lumen because of disturbance in oxidative protein folding leading to ER stress. A considerable amount of disturbance in the normal functioning of ER triggers an evolutionarily conserved response called unfolded protein response (UPR) resulting from the accumulation of unfolded proteins. The UPR enhances the protein folding capacity of the body by inducing the expression of genes and promoting ER-associated protein degradation to remove misfolded proteins (Schröder and Kaufman, 2005). However, mounting evidence suggests that chronic UPR activation leads to cell dysfunction and death with significant implications in the pathogenesis of endothelial dysfunction, a crucial feature of hypoxia-induced thrombosis (Migliacci et al., 2007).

Thrombosis is the formation of a blood clot or thrombus within the blood vessel that obstructs the flow of blood through the circulatory system causing serious health complications. It remains one of the major causes of morbidity and mortality among patients with cardiovascular conditions or those undergoing hip and knee replacement surgery. It is also a serious concern among the air travelers and mountaineers and to lowlanders stationed at extreme altitudes for the various work related activities (Rosendaal, 2002; Brill et al., 2013). A complex process like thrombus formation involves endothelial activation, inflammation, platelets aggregation and formation of cross-linked fibrin. All of these processes enhanced under the hypoxic condition are linked with the effects of sirtuins activity (Saha et al., 2011; Evans, 2019). The well-coordinated interactions between the activities of sirtuins and HIF, further implicate the therapeutic potential of sirtuins in the management of hypoxia-induced thrombosis. This review, therefore, discusses the potential role of an intersection of UPR and inflammation in the pathological manifestation of hypoxia-induced thrombosis and hypothesizes a new therapeutic role for sirtuins.

## Hypoxia-Induced Thrombosis and Sirtuins

Inadequate oxygen content in the ambient air may lead to a difference between tissue demand and oxygen supply resulting in a condition called hypoxia. The human cells require a continuous supply of O_2_ for various processes such as oxidative phosphorylation (OXPHOS) occurring during the electron transport chain in the inner mitochondrial membrane and disulfide bond formation during protein folding in ER. The human body undergoes a significant mechanism mediating adaptive response to hypoxia, activated by HIF-1 signaling to sustain cellular homeostasis in a low oxygen environment (Semenza, 2010). HIF-1 consists of an alpha (α)- and a beta (β)-subunits that makes it a heterodimeric transcription factor. The α-subunit has an oxygen-dependent degradation domain (Keith et al., 2011), which is hydroxylated by proline hydroxylase-2 (PHD-2) under the normoxic cellular conditions. The hydroxylation of the α-subunit as recognized by von-Hippel-Lindau-ubiquitin ligase complexes makes it vulnerable for proteasomal degradation (Huang et al., 1998). On the other hand, the β-subunit is constitutively expressed (Majmundar et al., 2010). Like PHD-2, another prolyl hydroxylase enzyme, factor inhibiting HIF-1 (FIH) also inactivates HIF-1 by hydroxylation of the α-subunit. Such post-translational modification averts the interaction of this α-subunit with the coactivators p300 and CREB binding protein, thereby suppressing the transcriptional activity of HIF-1. However, during hypoxia, PHDs and FIH become indolent due to undersupply of oxygen, sanctioning stabilization and activation of HIF-1 protein. Consequently, HIF-1 proteins are localized to the nucleus so that HIF-1 heterodimers can bind to hypoxia-response elements (HRE) present on the target genes for their transcriptional activation. As a transcription factor, HIF affects and regulates the expression of dozens of genes in response to change in oxygen concentrations that regulates energy metabolism, erythropoiesis, angiogenesis, and cell survival and restores oxygen homeostasis and maintains the health of endothelium (Yoon et al., 2006). For example, HIF-1 induced angiogenesis ensures the increased blood flow to the hypoxic tissues. The induction of erythropoietin (EPO) by HIF-1 enhances the oxygen-carrying capacity of the blood (Haase, 2013). However, continual hypoxia stimulus, as we discussed before, also drives several pathological pathways including those that are responsible for thrombotic phenotypes. Research studies have demonstrated the induction of the HIF-1 target genes due to the activation of the HIF-1 signaling pathway as the primary contributors of thrombus formation (Gupta et al., 2019). For example, endothelial HIF-1 and HIF-2 targets include genes such as tissue factor (TF) and plasminogen activator inhibitor (PAI)-1 that control coagulation (Liao et al., 2007).

**Figure 1:**
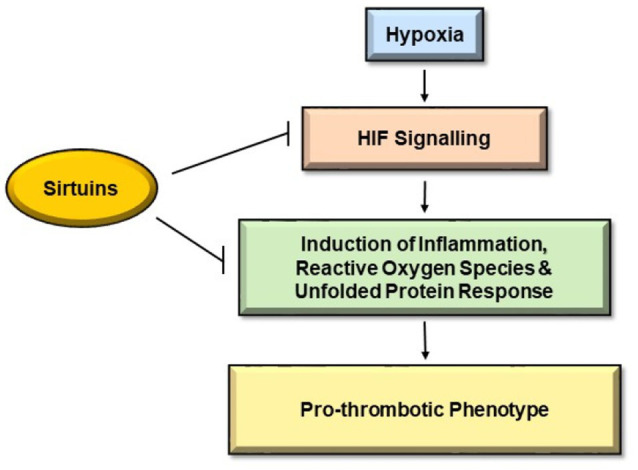
Pathways inhibited by Sirtuins to mitigate hypoxia induced thrombosis. Hypoxia causes endothelial dysfunction leading to activation of hypoxia-inducible factor (HIF) signaling pathways, which further leads to induction of inflammation, reactive oxygen species (ROS), and unfolded protein pathway (UPR). These pathways initiate coagulation cascade giving rise to pro-thrombotic phenotype. Sirtuins hamper HIF signaling pathways and hinder the induction of inflammation, ROS, and UPR, thereby indicating a protective role against hypoxia-induced thrombosis.

Sirtuins not only are key regulators of the cellular response to hypoxia (Finley et al., 2011) but are also identified in the amelioration of thrombotic manifestations (Breitenstein et al., 2011; Figure 1). Firstly, we will discuss the interaction of sirtuins with HIF-1 signaling followed by their role in attenuation of thrombus formation. A redox sensor, SIRT1, and a well-known O_2_ sensor, HIF-1α, are regulated in a well-coordinated manner during hypoxic conditions. A study demonstrated the repression of HIF-1α signaling by SIRT1 through deacetylation of HIF-1α at Lys674 residue (Lim et al., 2010). The same study also showed the inhibition of SIRT1 due to decreased NAD+ during hypoxia that consequently activated HIF-1α. They further associated SIRT1 with HIF-1α in the hypoxia-exposed tissues of mice that negatively regulated angiogenesis and tumor growth. SIRT6 again through its activity as an H3K9 deacetylase functions as a transcriptional co-repressor of HIF-1α (Zhong et al., 2010). SIRT7 is demonstrated as a negative regulator of HIF-1 and HIF-2 transcriptional activity independent of its deacetylase activity (Hubbi et al., 2013). The study also suggested the negative regulation of HIF-1α and HIF-2α protein by SIRT7 through an independent mechanism that does not involve prolyl hydroxylation and proteasomal degradation. In another study, mitochondrial SIRT3 has been demonstrated as a crucial regulator of the Warburg effect, which is characterized by high glycolysis even in the presence of oxygen. They found the overexpression of SIRT3 repressed glycolysis and proliferation of breast cancer cells. The group also suggested destabilization of HIF-1α activity by SIRT3 through its regulation of ROS production (Finley et al., 2011). Another similar study demonstrated the tumor suppressor property of SIRT3 through its ability to suppress ROS and regulation of HIF-1α (Bell et al., 2011). Thus, it seems clear that HIF activity is modulated by sirtuins. Nevertheless, mixed reports on the expression of sirtuins at low oxygen concentration persist. While one study observed a reduction of SIRT1 and SIRT4 at their protein levels under hypoxia (Pecher et al., 2020), few other studies demonstrated the increase in SIRT1 gene expression in a HIF-dependent manner during hypoxia (Chen et al., 2011). These conflicting results are suggestive of much more complex interactions between sirtuins and HIFs under hypoxia that are yet to be understood.

Furthermore, direct effects of sirtuins on the thrombus formations are known under normal oxygen concentrations. A study demonstrated the inhibition of platelet aggregation *in vitro* and pulmonary thrombus formation *in vivo via* downregulation of platelet activating factor receptor expression on platelets through SIRT1 (Kim et al., 2016). SIRT2 regulates platelet activity by acetylating and inhibiting protein kinase B (Akt), which participates in platelet aggregation, granule release and calcium transport (Moscardó et al., 2015). SIRT1 promotes endothelial-dependent vasodilation by targeting endothelial nitric oxide synthase (eNOS; Mattagajasingh et al., 2007). The eNOS-generated nitric oxide plays a crucial role in the inhibition of platelet aggregation, reduction of vascular smooth muscle proliferation, downregulation of leukocyte endothelium adhesion, chemokine expression, and inflammatory cell infiltration (Shimasaki et al., 2007). Another study observed the generation of occlusive thrombi in wild-type mice through an increased TF expression and inhibition of SIRT1. The same study observed that SIRT1 activation was sufficient to decrease the abnormal TF activity and alleviate the prothrombotic status in COX-2 knockout mice (Barbieri et al., 2012). One more study further demonstrated the *in vivo* elevation of experimental thrombosis by an increase of neutrophil extracellular traps (NETs) and TF because of a loss-of-function of SIRT3 (Gaul et al., 2018). Their study observed thrombo-protective effects of endogenous SIRT3 as lower expression levels of SIRT3 and SOD2 in CD14+ leukocytes were associated with acute coronary thrombosis in acute ST-elevation myocardial infarction patients. The group suggested usage of pan-sirtuin activating NAD+ boosters for enhancing sirtuins activity in the body that may provide a novel therapeutic target to prevent or treat thrombotic arterial occlusions. All of these observations in different studies together implicate the therapeutic potential of sirtuins in the alleviation of hypoxia-induced thrombosis.

## Implications of Unfolded Protein Response in Hypoxia-Induced Thrombosis and the Role of Sirtuins

**Figure 2:**
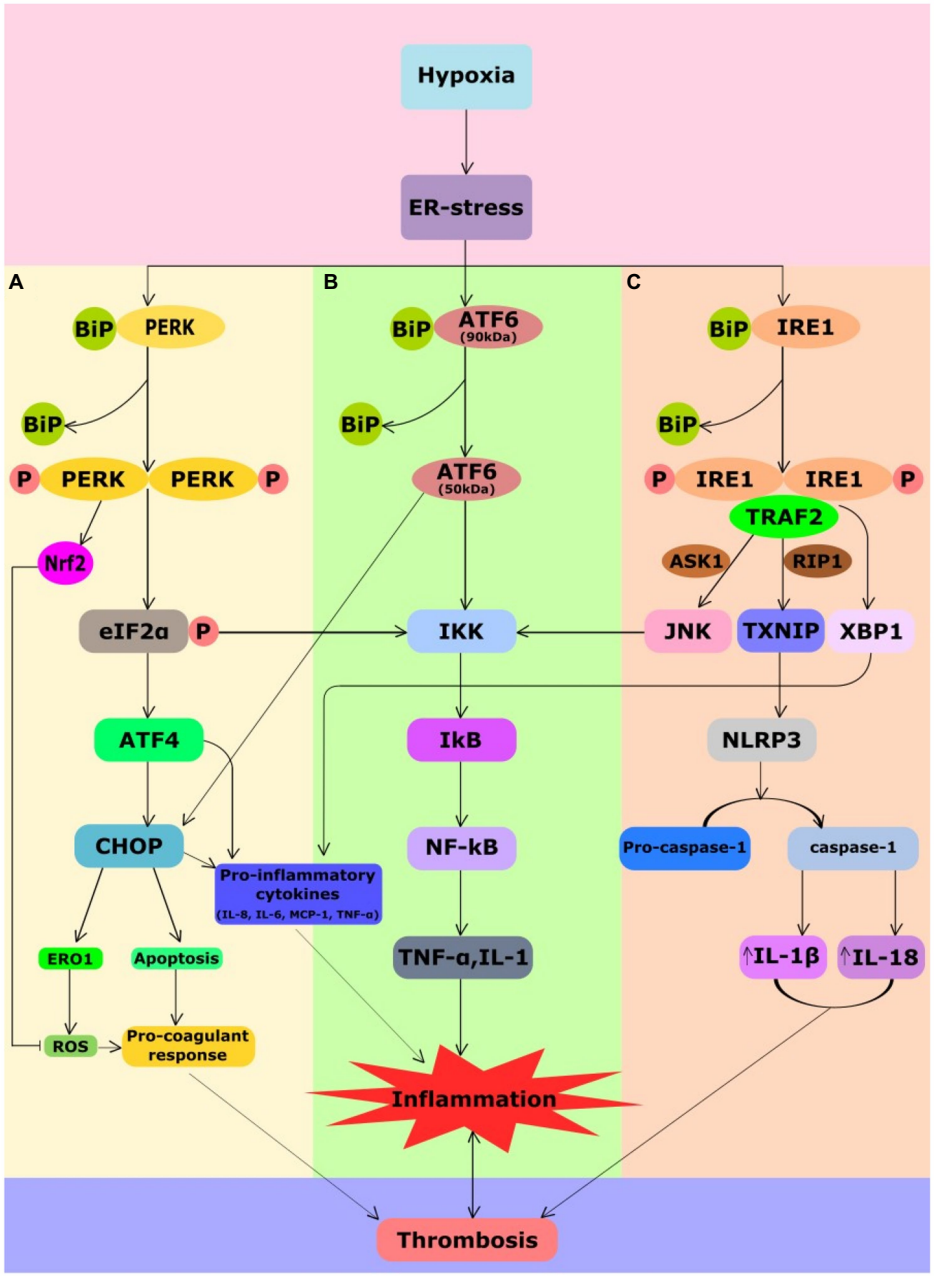
Crosstalk between UPR, inflammatory and coagulation pathways in hypoxia-induced thrombosis. Hypoxia causes ER stress that leads to activation of UPR signaling. UPR signaling comprises three sensor proteins PERK, IRE1, and ATF6. All the three branches of sensor proteins could cause inflammation. Under normal conditions, UPR sensor proteins are bound to ER chaperone, BiP, due to which these sensors remain inactivated. During ER stress, BiP dissociates from them leading to their activation. **(A)** Phosphorylation of eIF2a by PERK attenuates translation but selectively favors ATF4 translation. ATF4 favors CHOP expression that mediates generation of ROS, apoptosis, and release of pro-inflammatory cytokines promoting inflammation and pro-coagulant response. Although PERK-mediated Nrf2 has an antioxidant response, chronic ER stress can still lead to inflammation and pro-coagulant response. PERK-mediated attenuation of translation leads NF-kB translocation to the nucleus that regulates cytokine encoding TNF-α and IL-1 genes. **(B)** ATF6 branch of UPR also mediates expression of CHOP and NF-kB promoting inflammation. **(C)** Activated IRE1 splices XBP1 pre-mRNA to yield the spliced XBP1 mRNA to induce the production of the inflammatory cytokines IL-8, IL-6, MCP1, and TNF-α. IRE1 also induces elevation of thioredoxin-interacting protein (TXNIP), which activates the NLRP3 inflammasome that promotes elevation of IL-18 and IL-1β. Activated IRE1α also recruits TRAF2, which activates JNK and IKK leading to NF-kB expression. All of these processes potentiate venous thrombosis in response to hypoxia. ASK1, apoptosis signal-regulated kinase 1; ATF4, activating transcription factor 4; ATF6, activating transcription factor 6; BIP, binding immunoglobulin protein; CHOP, C/EBP homologous protein; ER, endoplasmic reticulum; ERO1, ER oxidoreductin 1; IKK, I kappa B Kinase; IRE1, inositol-requiring enzyme 1; IL-18, interleukin-18; IL-1B, interleukin-1β; JNK, c-Jun N-terminal kinase; NF-κB, nuclear factor κ-light-chain-enhancer of activated B cells; MCP-1, monocyte chemoattractant protein 1; Nrf2, nuclear factor erythroid 2-related factor-2; P, phosphorylation; PERK, protein kinase RNA-like ER kinase; RIP1, receptor-interacting serine/threonine-protein kinase 1; ROS, reactive oxygen species; TRAF2, tumor necrosis factor TNF receptor-associated factor-2; TXNIP, thioredoxin interacting protein; and XBP1, X-box binding protein 1.

The term “endoplasmic reticulum stress” defines any perturbation that compromises the protein folding functionality of the ER (Walter and Ron, 2011). The ER has a vital function of protein and lipid synthesis, lipid and glucose metabolism, detoxification of xenobiotics, maintaining calcium homeostasis, maturation and assembly of proteins and protein folding in the cell. The formation of disulfide bridges is a crucial process for ER protein homeostasis and is referred to as oxidative protein folding. In hypoxia, lack of O_2_ in cells contributes to the accumulation of misfolded proteins because of disturbance in oxidative protein folding leading to ER stress (Koumenis et al., 2007). Hence, a strong relationship exists between hypoxia and the accumulation of misfolded proteins in the ER, leading to activation of the UPR signaling pathway. The glucose-regulated protein of 78kDa (GRP78) or binding immunoglobulin protein (BiP) prevents aggregation of misfolded proteins within the ER and promotes folding by using the energy from ATP hydrolysis. Under normal conditions, UPR sensor proteins are bound to BiP to remain inactivated. When the misfolded proteins accumulate, BiP releases critical transmembrane ER stress sensor proteins like PERK, Inositol-requiring enzyme 1 (IRE1) and activating transcription factor 6 (ATF6) to launch UPR (Schröder and Kaufman, 2005). Figure 2 illustrates activation of UPR sensor proteins upon exposure to hypoxia. As depicted in Figure 2A, PERK, a type I trans-membrane kinase undergoes trans-auto-phosphorylation upon activation that inhibits the protein synthesis by eIF2α phosphorylation (Harding et al., 1999). PERK also facilitates hypoxia-induced translational attenuation (Koritzinsky et al., 2006). It represses translation of most mRNAs but selectively increases the translation of activating transcription factor (ATF4). Subsequently, ATF4 regulates the gene promoters involved in UPR and has been reported to be translationally upregulated under hypoxia in a PERK-dependent manner (Blais et al., 2004). Upon release from GRP78, ATF6 (90kDa) is transported to the Golgi body to be cleaved by S1P/S2P proteases to yield the active transcription factor ATF6 (50kDa) as shown in Figure 2B. Activated ATF6 directly modulates transcriptional induction of UPR target genes such as GRP78, ER-associated protein degradation, and C/EBP homologous protein (CHOP). Upon activation, IRE1 also undergoes trans-auto-phosphorylation to remove a 26 base pair intron yielding the spliced XBP1 mRNA (sXBP1; Figure 2C). A potent transcription activator, sXBP1, induces the expression of genes involved in the degradation of unfolded proteins and restoration of protein folding (Lee et al., 2003).

Nonetheless, UPR intersects with many stress-signaling pathways such as inflammatory pathways and apoptosis in endothelial cells and macrophages (Figure 2). These pathways involved in the upregulation of pro-inflammatory cytokines (Migliacci et al., 2007) are implicated in the aggravation of thrombosis pathogenesis. Under ER stress, PERK-ATF4 signaling favors expression of CHOP, which promotes release of inflammatory cytokines mediating inflammation, ROS and apoptosis mediating pro-coagulant response (Figure 2A). PERK facilitates NF-κB release to activate the expression of genes involved in downstream pathways of inflammation such as TNF-α and Interleukin-1 (IL-1; Deng et al., 2004; Hotamisligil, 2010). The ATF6 pathway also activates CHOP and the NF-κB pathways mediating inflammation (Yamazaki et al., 2009; Figure 2B). IRE1 induces elevation of thioredoxin-interacting protein (TXNIP), which activates the NLRP3 inflammasome (Lerner et al., 2012), a major player of inflammatory pathways (Figure 2C). Activated IRE1, additionally, recruits tumor necrosis factor (TNF) receptor-associated factor-2 (TRAF2), which interacts with c-Jun N-terminal kinase (JNK) and IκB kinase (IKK) to subsequently phosphorylates and starts the other downstream inflammatory pathways (Hu et al., 2006). Furthermore, XBP1s and ATF4, both induce inflammatory cytokines like IL-8, IL-6, monocyte chemoattractant protein-1 (MCP1), and TNF-α in the human endothelial cells (Hotamisligil, 2010). Under ER stress, the release of calcium and the production of mitochondrial ROS increases the generation of intracellular ROS (Laurindo et al., 2012). ER-localized ROS can be a by-product of endoplasmic oxidoreductin 1 (ERO1) during oxidative protein folding (Kaludercic et al., 2014) or of ER-localized NADPH oxidases (BelAiba et al., 2007). Prolonged ER stress also activates pro-apoptotic pathways in atherosclerosis and cardiovascular diseases (CVDs; Tabas, 2010; Choy et al., 2018). CHOP and IRE-1 are involved in the ER stress-related apoptotic signals (Myoishi et al., 2007; Hong et al., 2017). IRE1 and TRAF2 complex, closely related to the signal transduction factor apoptosis signal-regulating kinase (ASK)-1, activates JNK and then regulates Bcl-2 family members to promote cell apoptosis (Nishitoh et al., 2002; Tabas, 2010). In addition, the interactions of IRE1α/TRAF2/ASK1 activate caspase-12 and eventually induce apoptosis (Hong et al., 2017). Under high ER stress signals, the IRE1-dependent degradation of ER-related mRNAs through regulated IRE1α-dependent decay (RIDD) also leads to cell apoptosis (Hollien et al., 2009; Tabas and Ron, 2011). The occurrence of phosphatidylserine (PS) is one of the hallmarks of cells undergoing apoptosis (Fadok et al., 1992), whose exposure at the surface of apoptotic lymphocytic, monocytic, smooth muscle, or endothelial cells can induce pro-coagulant response (Aupeix et al., 1996, 1997; Bombeli et al., 1997; Flynn et al., 1997). PS promotes the surface assembly and the catalytic efficiency of the characteristic enzyme complexes of the blood coagulation cascade (Pei et al., 1993), including the TF/factor VIIa complex (Bach and Rifkin, 1990). Intrinsic apoptotic pathways that are eventually triggered further lead to cell death in persistent ER stress (Hetz, 2012). A recent study has associated UPR signaling in the prothrombotic transformation of pancreatic cancer (Muse et al., 2019). Their study indicated that TF-bearing extracellular vesicles formation were upregulated by UPR-mediated vesicular trafficking. The group further demonstrated the presence of significant upregulation of UPR markers in the plasma samples of pancreatic cancer patients, who developed clots compared to the plasma samples of those with no clot. Under pathological conditions, TF is expressed on microvesicles and circulating leukocytes activating endothelial cells. These sources of TF act as a source of “blood clot” (Mackman, 2004) that are activators of the extrinsic coagulation system and trigger both arterial and venous thrombosis (Grover and Mackman, 2018). Though a direct link between UPR and thrombus formation still needs major exploration. Nonetheless, linking of ER stress with major pathological pathways that play a definitive role in the thrombus formation is suggestive of the pathophysiological potential of UPR in various thrombotic manifestations.

Sirtuins are majorly involved in the attenuation of ER stress-mediated pathways. SIRT1 protects cardiomyocytes from ER stress-induced apoptosis by attenuating PERK/eIF2α pathway activation (Prola et al., 2017). As a downstream transcriptional factor of PERK/eIF2α/ATF4, CHOP plays a critical role in ER stress-induced apoptosis through suppression of the anti-apoptotic protein Bcl-2 and induction of the pro-apoptotic molecules such as the pro-apoptotic Bcl-2 family member, death receptor 5, and telomere repeat binding factor 3 (Cao, 2015). SIRT1 is demonstrated to reduce apoptosis by downregulating the expression of CHOP and caspase-12, which are key molecules in ER stress-mediated apoptotic pathways (Aoyama et al., 2005; Ren et al., 2019). Further, sirtuins deacetylate XBP1s that could prevent ER stress-mediated release of pro-inflammatory cytokines and apoptosis (Wang et al., 2011). XBP1s are also deacetylation targets of SIRT6 against ER stress-induced hepatic steatosis (Bang et al., 2019). Along with SIRT6, XBP1 also induces SIRT7 expression, which reduces ER stress induced protein expressions, such as CHOP, XBP1s, and GRP78 (Shin et al., 2013). In a rat diabetic model, SIRT3 protects pancreatic β-cells from ER stress-mediated apoptosis through calcium influx inhibition and the hyper acetylation of GRP78 (Zhang et al., 2016). The study showed SIRT3 knockdown effectively enhanced the upregulation of PERK, IRE1, ATF6, and CHOP induced by palmitate, and promoted palmitate-induced β-cell apoptosis and dysfunction. Additionally, in the cardiomyocytes derived from a diabetic rat model demonstrated the significant restoration of cardiac function, reduction in cardiomyocyte apoptosis, and amelioration of ER stress by a SIRT1 activator, resveratrol (Guo et al., 2015). In the interscapular brown adipose tissue of high-fat diet-induced obese mice, SIRT1 overexpression alleviated ER stress-induced brown adipocyte apoptosis by inhibiting Smad3 and ATF4 (Liu et al., 2017). All these studies discussing the protective role of sirtuins in the reduction of ER stress response additionally hints at the benefits of sirtuins as therapeutic prevention for hypoxia-induced thrombosis.

## Implications of Inflammatory Pathways in Hypoxia-Induced Thrombosis and the Role of Sirtuins

The activation of multiple cell types such as endothelium, platelets, and lymphocytes indicates a strong link between hypoxia response and inflammation (Mannucci et al., 2002; Eltzschig and Carmeliet, 2011). Hypoxia-induced inflammation causes hemostatic imbalance leading to endothelial and platelet activations, which further leads to the secretion of pro-thrombotic and anti-fibrinolytic factors. Prolonged hypoxia is associated with increased mitochondrial ROS generation leading to oxidative stress (Zorov et al., 2014). Several other sources such as NADPH oxidase (Marshall et al., 1996) or xanthine oxidase (Abramov et al., 2007) are involved in the modulation of ROS levels under hypoxia. ROS increases the expression of TF in endothelial cells (Golino et al., 1996), monocytes (Cadroy et al., 2000) and vascular smooth muscle cells (Herkert et al., 2002) to stimulate clotting. In addition, ROS oxidatively modified proteins involved in coagulation may favor a procoagulant state. The only physiologic regulator of TF activity, TF pathway inhibitor (TFPI), can be inhibited by oxidative stress, and exert a procoagulant effect (Ohkura et al., 2004). ROS can also directly inactivate protein C (Nalian and Iakhiaev, 2008) and its upstream agonist thrombomodulin that are major anticoagulant proteins (Glaser et al., 1992). ROS regulates P-selectin by interaction with P-selectin glycoprotein ligand-1 on neutrophils (Etulain et al., 2015), associated with an increased risk of venous thromboembolism (Ay et al., 2007). The upregulation of NF-κB is closely linked with ROS generation in inflammation and obesity (Tornatore et al., 2012). Thus, a cross-talk between coagulation proteins and inflammatory molecules has emerged as a critical molecular event toward the onset of thrombotic events. Interestingly, studies from our lab explored that hypoxia accelerated thromboembolic events by stimulating the activation of NLRP3 inflammasome complex to upregulate (IL)-1β secretion (Gupta et al., 2017). The cytokine IL-1β activates endothelial cells, dysfunctions vascular smooth muscle cells, and induces pro-coagulant and adhesion protein expression (Bevilacqua et al., 1984). It also influences the NETs formation, whose association with TF triggers the coagulation cascade in atherothrombosis (Liberale et al., 2019). These NETs can enhance coagulation through the upregulation of platelet aggregation, red blood cells recruitment, and fibrin deposition (Fuchs et al., 2010). They can also cause endothelial activation and damage (Gupta et al., 2010). Activated leukocytes or neutrophils can also release NETs, consisting of decondensed chromatin and DNA (Fuchs et al., 2010).

Further inflammatory cytokines trigger surface expression of adhesion receptors on endothelial cells that facilitates binding of circulatory leukocytes and microvesicles (Esmon, 2008). Activated platelets release soluble mediators such as chemokines, which support the recruitment of interleukins that potentiates inflammation. Cytokines such as IL-1β and TNF-α derived from activated leukocytes during inflammation induce TF expression on the endothelium surface favoring thrombin formation (Esmon, 2008). The activation of a crucial modulator of inflammation and thrombosis, NF-κB, stabilizes HIF-1α in hypoxia (van Uden et al., 2008). Further, endothelial cells contain Weibel-Palade bodies (WPB) storing von Willebrand factor (vWF) and P-selectin molecules that are thrombosis- and inflammation-related constituents (Wagner et al., 1982; McEver et al., 1989). Hypoxic condition is a known inducer of secretions from WPB (Pinsky et al., 1996). vWF binds to receptors glycoprotein-Ibα and glycoprotein-IIb-IIIa on the platelet surface to mediate platelet adhesion and recruitment (Bergmeier et al., 2008). In response to oxidative stress, Nrf2, which is activated in PERK dependent manner, activates various antioxidant responsive element (ARE)-dependent genes (Kensler et al., 2007). Under normal physiological conditions, Nrf2 is bound to Kelch-like ECH-associated protein 1 (KEAP1); however, on exposure to oxidative stress, it disassociates from KEAP1 and translocates into the nucleus to upregulate the transcription of antioxidant genes (Sajadimajd and Khazaei, 2018).

Upregulating levels of SIRT1 increase the nuclear translocation of Nrf2 to mitigate the cellular damage caused by oxidative stress and inhibits mitogen-activated protein kinase phosphorylation (Ding et al., 2016; Gu et al., 2016). By activating the FOXO3 transcription factor, SIRT1 has an antioxidative effect *via* modulations in SOD2 and CAT (Brunet et al., 2004; Wang et al., 2012). SOD2 converts superoxide anion radicals to hydrogen peroxide (H_2_O_2_), and molecular oxygen (O_2_) and CAT converts H_2_O_2_ to H_2_O and O_2_. In response to oxidative stress, SIRT2 deacetylates FOXO3a, a transcriptional activator of the SOD2 gene that encodes a powerful antioxidant SOD2 protein (Wang et al., 2012). The other targets of SIRT2 such as G6PD, PGAM2, and NF-κB also function in ROS mediation (Gomes et al., 2015). G6PD regenerates GSH from its oxidized form. PGAM2 results in the increased production of NADPH required for GSH production and NF-κB targets antioxidant genes such as SOD2 (Kairisalo et al., 2007). SIRT3 also controls systemic levels of oxidative stress in response to hypoxia by acting on the FOXO3/SOD2 signaling pathway (Brunet et al., 2004). SIRT3 influences the production of ROS through the modulation of enzymes involved in the mitochondrial OXPHOS pathway to affect cellular health directly (Haigis et al., 2012). SIRT3 activates important enzymes such as SOD2, CAT, and isocitrate dehydrogenase (IDH) in reducing the cellular burden of ROS (Merksamer et al., 2013; Tao et al., 2014). SIRT4 may play an essential role in the regulation of the antioxidant response by managing the players involved in it (Singh et al., 2018). SIRT5 increases the activity of SOD1 after desuccinylation (Lin et al., 2013). It protects cardiomyocytes from oxidatively induced apoptosis, although, the mechanism by which this protection occurs is unclear (Liu et al., 2013). SIRT6 overexpression has been shown in cardiomyocytes as protection against ischemia-reperfusion injury by the oxidative stress reduction and endogenous antioxidants upregulation *via* an energy-sensing kinase AMPK-FOXO3α axis necessary for oxidative stress resistance (Wang et al., 2016). SIRT6 co-activates Nrf2 in the human mesenchymal stem cells (hMSCs) as a protection against oxidative stress (Pan et al., 2016). SIRT1 inhibits the NF-κB signaling directly by deacetylating the p65 subunit of NF-κB complex (Kauppinen et al., 2013). SIRT1 and SIRT2 regulate acetylation of a master regulator of mitochondrial biogenesis, PGC-1α that has been associated with a reduction in ROS levels and upregulation of antioxidant enzyme expression (Nemoto et al., 2005; Krishnan et al., 2012). Inhibition of SIRT1 induces arterial thrombus formation *in vivo* through induction of TF expression and activity by increasing NFκB/p65 activation in the human endothelial cells (Breitenstein et al., 2011). SIRT1 and SIRT2 inhibit the NF-κB signaling directly by deacetylating the p65 subunit of NF-κB complex (Rothgiesser et al., 2010; Kauppinen et al., 2013). SIRT6 deacetylates histone H3K9 at the chromatin level to attenuate NF-κB signaling (Kawahara et al., 2009). SIRT1 inhibits NLRP3 inflammasome activation and subsequent IL-1β secretion (Li et al., 2017) and downregulates many pro-inflammatory molecules such as interleukins, TNF-α, and MCP-1 (Shen et al., 2009; Schug et al., 2010). Hence, sirtuins have a pre-eminent role in altering oxidative stress suggestive of its therapeutic potentials in inflammation and hypoxia-induced thrombosis.

## Future Perspectives and Conclusion

Hypoxia in cells has numerous effects such as upregulation of ER-stress mediated oxidative stress, inflammation-related pathologies, and loss of endothelium integrity. All of these lead to thrombotic phenotypes. It thus becomes imperative to relieve these pathologies in the cells. Sirtuins are a competent therapeutic molecule proven protective in oxidative and ER stress-induced pathways such as inflammation and apoptosis (Hotamisligil, 2010; Tabas, 2010). Aqueous extract of Whitmania Pigra, a traditional Chinese medicine to treat cardiovascular diseases, has demonstrated to reduce deep vein thrombosis burden by inhibiting inflammation *via* SIRT1/NF-κB signaling pathway (Yao et al., 2020). Potent pharmacological Sirtuins activators like Sirtuins activating compounds (STAC) that increase the Sirtuins activity are available and few of them are currently under clinical trials in various diseases. Resveratrol, a natural STAC, is known to activate SIRT1 and affect SIRT3 and SIRT5 (van der Meer et al., 2015). Resveratrol has anti-inflammatory, anticoagulatory, and anti-fibrinolytic effects in the cell culture. It decreases vWF, tPA-1, and IL-8 secretions in HUVECs cells (Shahidi et al., 2020). Few other SIRT1 artificial activators, SRT2104, SRT2379, and SRT3025, are under clinical trials (van der Meer et al., 2015). SRT2104 is currently under examination in healthy volunteers (Dai et al., 2018). It reduces the LPS-induced release of IL-6 and IL-8 but not TNFα or IL-10 and inhibits LPS activation of coagulation. Further, sirtuins have shown promise in their ability to improve the health of various cardiovascular and metabolic disease patients (Kane and Sinclair, 2018). Discussion of sirtuin modulators potential in cancer treatment is also undertaken (Yuan et al., 2013). Therefore, the physiological functions of sirtuins in alleviating thrombosis and the clinical studies highlighting their therapeutic potential is adequate to indicate a new beneficial role for sirtuins in hypoxia-induced thrombosis.

## Author Contributions

KS contributed in the writing and organizing of the manuscript. MA contributed in the writing and edited the manuscript. AM contributed in the writing and supervised and edited the manuscript. All authors contributed to the article and approved the submitted version.

## Funding

This work was supported by the Science and Engineering Research Board-Department of Science and Technology, Government of India (Project code: SB/WEA-02/2019) and Council of Scientific and Industrial Research, India.

## Conflict of Interest

The authors declare that the research was conducted in the absence of any commercial or financial relationships that could be construed as a potential conflict of interest.

## Publisher’s Note

All claims expressed in this article are solely those of the authors and do not necessarily represent those of their affiliated organizations, or those of the publisher, the editors and the reviewers. Any product that may be evaluated in this article, or claim that may be made by its manufacturer, is not guaranteed or endorsed by the publisher.

## Figures and Tables

**Table:** 

ATF6	Activating transcription factor 6
ATF4	Activating transcription factor
ARE	Antioxidant responsive element
ASK-1	Apoptosis signal-regulating kinase
BiP	Binding immunoglobulin protein
CVDs	Cardiovascular diseases
CHOP	C/EBP homologous protein
eIF2α	Eukaryotic translation initiation factor 2α
eNOS	Endothelial nitric oxide synthase
EPO	Erythropoietin
ERO1	Endoplasmic oxidoreductin 1
FIH	Factor Inhibiting HIF-1
FOXO	Forkhead box protein
G6PD	Glucose 6-phosphate dehydrogenase
GSH	Glutathione
GRP78	Glucose-regulated protein of 78kDa
HIF-1	Hypoxia-inducible factor 1
HREs	Hypoxia-response elements
IKK	IκB kinase
IRE1	Inositol-requiring enzyme 1
IDH	Isocitrate dehydrogenase
JNK	c-Jun N-terminal kinase
KEAP1	Kelch-like ECH-associated protein 1
LPS	lipopolysaccharide
MCP-1	Monocyte chemoattractant protein 1
NAD+	Nicotinamide adenine dinucleotide
NETs	Neutrophil extracellular traps
NF-κB	Nuclear factor κ-light-chain-enhancer of activated B cells
Nrf2	Nuclear factor erythroid 2-related factor-2
PAFR	Platelet activating factor receptor
PAI	Plasminogen activator inhibitor
PERK	Protein kinase RNA-like endoplasmic reticulum kinase
PGC-1α	Peroxisome proliferator-activated receptor gamma coactivator 1-alpha
PHD-2	Proline-hydroxylase-2
PS	Phosphatidylserine
RIDD	Regulated IRE1α-dependent decay
ROS	Reactive oxygen species
SIRT	Sirtuins
SOD	Superoxide dismutase
STAC	Sirtuins activating compounds
TF	Tissue factor
TFPI	Tissue factor pathway inhibitor
TNF	Tumor necrosis factor
TRAF2	Tumor necrosis factor TNF receptor-associated factor-2
TXNIP	Thioredoxin-interacting protein
UPR	Unfolded protein response
VEGF	Vascular endothelial growth factor
VT	Venous thrombosis
VWF	Von Willebrand factor
WPB	Weibel-Palade bodies
XBP1	X-Box Binding Protein 1
